# P-498. Evaluating DoxyPEP's Role in Reducing Sexually Transmitted Infections among HIV Patients: A Two-Year Study at the HEAT Clinic

**DOI:** 10.1093/ofid/ofaf695.713

**Published:** 2026-01-11

**Authors:** Wefag Ahmed, Rachelle Thompson, Sara Abdelrahaman

**Affiliations:** SUNY Downstate Health Sciences University, peroria, IL; SUNY Downstate Health Sciences University, Brooklyn, New York; SUNY Downstate Health Sciences University, peroria, IL

## Abstract

**Background:**

Sexually transmitted infections (STIs) remain a public health issue, particularly in individuals living with HIV (PLHIV). Although doxycycline post-exposure prophylaxis (DoxyPEP) has been reported to reduce bacterial STIs in high-risk individuals, the use acceptability of DoxyPEP as an option in HIV-infected adolescents has been under mostly researched.

**Methods:**

A two-year retrospective cohort analysis was conducted on 91 HIV-infected patients at the HEAT Clinic in Brooklyn. Use of DoxyPEP and STI follow-up outcomes were ascertained by detailed clinical record abstraction. Patients were stratified by DoxyPEP use, demographics, and other relevant factors. Multivariate logistic regression was used to assess the association between DoxyPEP use and STI incidence while adjusting for potential confounders.

**Results:**

DoxyPEP use was highest in transgender women (100%) and males (58.8%), and none among cisgender women. Baseline STI risk was higher among those who started DoxyPEP (men: 80%, transgender women: 66.7%). Following implementation, 78.3% of DoxyPEP users and 28.1% of non-DoxyPEP users were diagnosed with at least one STI. Among DoxyPEP users, the following percentages of individual STIs were gonorrhea (20.6%), chlamydia (6.1%), and syphilis (16.7%). These findings indicate DoxyPEP users were at high risk and in need of specifically targeted prevention interventions.

**Conclusion:**

Increased DoxyPEP use was associated with decreased incidence of STIs among HIV-positive youth and young adults. The findings support DoxyPEP as part of standard care for this population in urban HIV clinics. Future studies should evaluate adherence, antimicrobial resistance, and implementation in women and non-MSM groups.

**Disclosures:**

All Authors: No reported disclosures

